# Development and Vibration Control of Frequency Adjustable Tuned Mass Damper Based on Magnetorheological Elastomer

**DOI:** 10.3390/ma15051829

**Published:** 2022-02-28

**Authors:** Jiarui Zhang, Yaoyang Zhu, Jianwei Tu, Zhao Li, Qiankun Wang

**Affiliations:** 1 Hubei Key Laboratory of Roadway Bridge and Structure Engineering, Wuhan University of Technology, Wuhan 430070, China; ZJR1207916099@whut.edu.cn (J.Z.); yaoyang@whut.edu.cn (Y.Z.); zhaoli_whut@whut.edu.cn (Z.L.); 2 School of Civil Engineering and Architecture, Wuhan University of Technology, Wuhan 430070, China

**Keywords:** magnetorheological elastomer, frequency adjustable tuned mass damper, Hilbert–Huang transform, natural excitation technique, real-time frequency tracking

## Abstract

Tuned mass dampers (TMD) have been widely used in passive vibration control, but their main disadvantage is that the vibration reduction effect may be greatly affected by the natural frequency of the main structure. In order to solve this limitation, we designed a frequency adjustable tuned mass damper (FATMD) based on a magneto rheological elastomer (MRE), which is a new type of magneto rheological smart material, with adjustable stiffness, obtained by changing the magnetic induction. We used MRE to change the stiffness of FATMD to track the natural frequency of the main structure. However, adding TMD will change the natural frequency of the system. Therefore, we combined Hilbert–Huang transform (HHT) and a natural excitation technique (NExT), with Simulink/dSPACE, to identify the natural frequency of the system in real time, and then calculated the natural frequency of the main structure through the TMD optimal design theory. This can help adjust FATMD to its optimum tuning state. To verify the applicability and effectiveness of FATMD, this paper compares the FATMD and traditional TMD experimental results. The natural frequency of steel beams can be changed by adding mass blocks. The experimental results indicate that FATMD, using the frequency tracking method, can effectively track the natural frequency of the main structure to ensure that the system is always in the optimum tuning state. In addition, FATMD can still achieve a good vibration reduction effect when the natural frequency of the main structure changes.

## 1. Introduction

The tuned mass damper (TMD) is composed of a mass, a stiffness element and a damping element, which can absorb the vibration energy of the main structure through its own vibration, to reduce the vibration response of the main structure. It has the advantages of simple structure, convenient layout, and effective vibration reduction effect. Nowadays, TMD has been widely used in civil engineering, such as Sydney Tower in Australia [1], CN Tower in Toronto, Taipei 101 Building [2], London’s Millennium footbridge [3,4], Pedro and Inês footbridge [5].

However, TMD also has the disadvantage of being sensitive to the natural frequency change of the main structure [6,7]. When the natural frequency of TMD deviates from the natural frequency of the main structure, that is, when the TMD system is detuned, its vibration reduction effect will be significantly reduced [8]. Rana et al. [9] found that when the main structure damping is low, the detuning of the TMD system has a greater impact on the peak and RMS value of the displacement response of the main structure. Wang et al. [10] studied the vibration control of high-speed railway bridges using TMD. The influence of a train on the natural frequency of the main structure was considered. The research results showed that when a train is running, the TMD can improve the local peak imbalance of the frequency domain response curve and reduce the vibration effectively, considering the change of the natural frequency of the structure. Okhovat et al. [11] took Tehran Tower as a case study, to investigate the effects of the system detuning on TMD performance, and found that when the natural frequency of the TMD deviates from the optimal design frequency, the vibration reduction effect will be significantly reduced.

On the other hand, the natural frequency of the main structure may vary at different stages. For example, the mass of the main structure changes during the construction process [12], the change of water level in an elevated storage tank, and the change of live load on a bridge will also change the natural frequency of the main structure [13,14]. Changes in structural natural frequency are not only affected by changes in mass, but also by degradation of structural stiffness. A sudden seismic load and the long-term repeated vehicle load will cause the stiffness of the structure to degrade [15,16,17]. In summary, the natural frequency changes of the main structure are quite common; however, the traditional TMD is unable to follow the natural frequency changes of the main structure, resulting in detuning. Therefore, it is necessary to develop a frequency adjustable tuned mass damper (FATMD), in order to adapt to the natural frequency changes of the main structure.

FATMD can be adjusted to the natural frequency of TMD by changing its stiffness or mass [18,19,20,21,22]. Chifley Tower [23], Taipei 101 [24] and Shanghai Tower [25] were adjusted to the natural frequency of TMD by artificially changing the pendulum length of the pendulum TMD, but this artificial adjustment method is to make the TMD meet the expected design requirements in the installation process, and cannot complete the real-time adjustment of the TMD in the working process. To realize the real-time adjustment of the natural frequency of TMD, Sun et al. [26] identified the natural frequency of the main structure by short-time Fourier transform (SFT) [27], so that the natural frequency of FATMD matches the natural frequency of the main structure. It was found that the SFT identification method can effectively observe the frequency change of the main structure and improve the vibration reduction effect. Shi et al. [28,29] developed a mass-changing FATMD, which pumped water from the bottom water tank of the TMD to the upper water tank of the TMD through a pump to change the mass of the TMD. The tuning method, based on the acceleration ratio between the main structure and the TMD, and the frequency identification method, based on SFT, were used to adjust the natural frequency of the TMD. It is beneficial to adjust the frequency of TMD by changing the mass, but the method of installing water pumps and tanks on TMD is cumbersome and difficult to achieve.

Magneto rheological elastomer (MRE) is a type of magneto rheological smart material that uses polymer elastomer as the matrix and contains magnetic particles [30,31]. It can quickly change its storage modulus with the change of the external magnetic field, and has the ability to change stiffness in real time [32]. As a stiffness adjustable element, MRE has been used in many applications, such as stiffness-adjustable spring elements [33], stiffness-adjustable differential mounts of vehicles [34] and Mechanical leg, using MRE joints [35]. It can be seen that MRE has a good stiffness adjustment ability and can be used to make intelligent vibration reduction devices.

Due to the fact that the MRE-based intelligent vibration reduction device is different from the passive control device, different control algorithms were needed to adjust the MRE stiffness. Ginder et al. [36] first designed a dynamic vibration absorber (DVA) based on MRE, and tested its performance. It was found that the DVA based on MRE has the advantages of high bandwidth and fast response. Komatsuzaki et al. [37,38] and Park et al. [39] proposed a DVA stiffness switching algorithm based on an external excitation frequency and an optimal method of MRE-based DVA based on a model-free reinforcement learning method, respectively, and both had good vibration reduction effect. Guan et al. [40] designed a DVA with MRE isolator, and built a vibration control system based on the limited sliding algorithm of linear quadratic optimal theory. Taking the five-story benchmark building model as an example, the numerical simulation of semi-active control was completed, and good vibration reduction effect was achieved. In the above research, appropriate control methods, based on the vibration characteristics of the structure and the external excitation frequency, are applied to make the DVA achieve the optimal vibration reduction effect, while the optimal design theory of TMD is not applied.

In the study of optimal design of adjustable TMD, based on MRE, Yang and Wang et al. [41,42] proposed a vibration reduction system for adjusting TMD stiffness through the MRE isolator. The system was used for the vibration control of the constructing bridge tower. In the vibration control simulation, the measurement data were analyzed by the system identification method to obtain the natural frequency of the bridge tower in the construction process and optimize the TMD. However, this method cannot achieve real-time adjustment, and it needs to artificially adjust the applied magnetic field. Therefore, FATMD based on MRE should adjust its natural frequency, according to the natural frequency of the main structure in real time, and ensure that the main structure is always in the optimal tuning state. Therefore, our research group proposed a real-time frequency identification method, based on Hilbert–Huang transform (HHT) and natural excitation technique (NExT), to identify the instantaneous natural frequency of the main structure in 2015 [43]. Further, HHT [44,45,46] can divide the signal into multiple intrinsic mode functions (IMFs) through empirical mode decomposition (EMD), and NExT [47,48] can eliminate the end effect of IMFs and convert them into free attenuation signals, and then obtain the instantaneous frequencies of different IMFs through Hilbert transform (HT). This identification method is easy to implement, which does not need to select the window function and is suitable for random load excitation, but the identified instantaneous frequency is the natural frequency of the main structure-FATMD system, rather than the natural frequency of the main structure itself. To solve this problem, we improved the frequency identification method. Through the TMD optimal design theory, the natural frequency of the system is converted into the natural frequency of the main structure, and the signal is segmented to realize the real-time refresh of the natural frequency of the main structure. Then, the natural frequency of the main structure itself can be calculated by the TMD optimal design theory. On this basis, we establish a functional relationship between the natural frequency of the main structure and the input current of the FATMD. By adjusting the current, FATMD can track the natural frequency of the main structure in real time to ensure that the FATMD vibration reduction system is always kept in an optimal tuning state.

## 2. Mechanism and Design of Frequency Adjustable Tuned Mass Damper (FATMD)

### 2.1. The Mechanism of Traditional Tuned Mass Damper (TMD)

**Figure 1:**
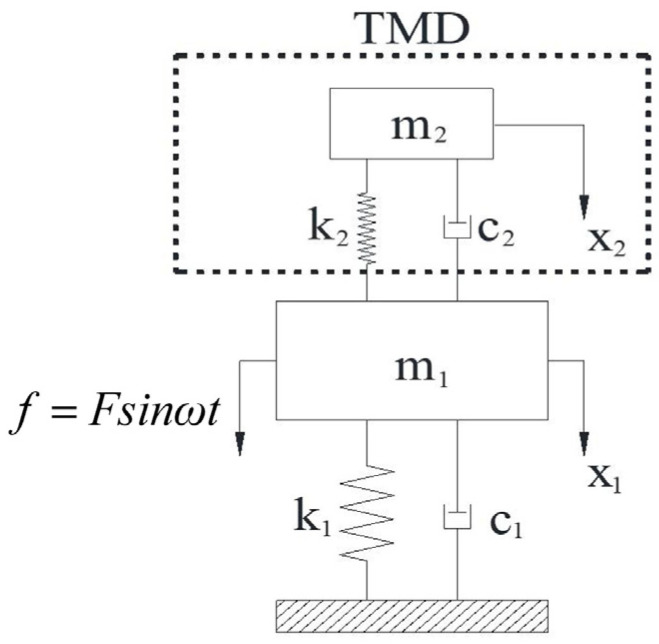
TMD-main structure system (TMD: tuned mass damper).

The traditional TMD is a passive vibration reduction device, composed of springs, mass elements and damping elements, as shown in Figure 1.

Under the action of external excitation, f=Fsinωt, the dynamic equation (Equation (1)) of the system is shown as below:

(1) $$\left[\begin{array}{cc} m_{1} & 0\\ 0 & m_{2} \end{array}\right]\left[\begin{array}{c} {\ddot{x}}_{1}\\ {\ddot{x}}_{2} \end{array}\right]+\left[\begin{array}{cc} c_{1}+c_{2} & -c_{2}\\ -c_{2} & c_{2} \end{array}\right]\left[\begin{array}{c} {\dot{x}}_{1}\\ {\dot{x}}_{2} \end{array}\right]+\left[\begin{array}{cc} k_{1}+k_{2} & -k_{2}\\ -k_{2} & k_{2} \end{array}\right]\left[\begin{array}{c} x_{1}\\ x_{2} \end{array}\right]=\left[\begin{array}{c} f\\ 0 \end{array}\right]$$

where $m_{1}$ and $m_{2}$ are the masses of the main structure and TMD; $k_{1}$ and $k_{2}$ are the stiffness of the main structure and TMD; $c_{1}$ and $c_{2}$ are the damping coefficients of the main structure and TMD; $x_{1}$, ${\dot{x}}_{1}$ and ${\ddot{x}}_{1}$ are the displacement, velocity and acceleration of the main structure; $x_{2}$, ${\dot{x}}_{2}$ and ${\ddot{x}}_{2}$ are the displacement, velocity and acceleration of TMD.

When the damping of the main structure is low, according to the TMD optimal design theory [49], the optimal damping ratio, the optimal frequency ratio design formula of TMD, and the acceleration amplitude ratio formula can be obtained, as follows:

(2) $$\xi_{opt}=\sqrt{\frac{3\mu }{4\left(1+\mu \right)\left(2+\mu \right)}}$$

(3) $$\gamma_{opt}=\frac{\omega_{2}}{\omega_{1}}=\frac{1}{\sqrt{1+\mu }}$$

(4) $$\frac{{\ddot{x}}_{1}}{{\ddot{x}}_{st}}=\sqrt{\frac{\lambda^{4}\left({\left(\gamma^{2}-\lambda^{2}\right)}^{2}+{\left(2\xi \lambda \right)}^{2}\right)}{\left({\left(\left(1-\lambda^{2}\right)\left(\gamma^{2}-\lambda^{2}\right)-\mu \gamma^{2}\lambda^{2}\right)}^{2}+{\left(1-\left(1+\mu \right)\lambda^{2}\right)}^{2}{\left(2\xi \lambda \right)}^{2}\right)}}$$

(5) $$\omega_{1}=\sqrt{\frac{k_{1}}{m_{1}}},\omega_{2}=\sqrt{\frac{k_{2}}{m_{2}}},\mu =\frac{m_{2}}{m_{1}},\lambda =\frac{\omega }{\omega_{1}},\gamma =\frac{\omega_{2}}{\omega_{1}},\xi =\frac{c_{2}}{2m_{2}\omega_{1}},X_{st}=\frac{f}{k_{1}}$$

where $\xi_{opt}$ and $\gamma_{opt}$ are the optimal damping ratio and the optimal frequency ratio of the TMD vibration reduction system; μ is the mass ratio of the TMD and the main structure; λ is the forced vibration frequency ratio; γ is the natural frequency ratio; ξ is the damping ratio; $\omega_{1}$ is the natural circular frequency of the main structure; $\omega_{2}$ is the natural circular frequency of TMD; ${\ddot{x}}_{st}$ is the static deflection of the main structure.

### 2.2. The Mechanism and Structure of FATMD

**Figure 2:**
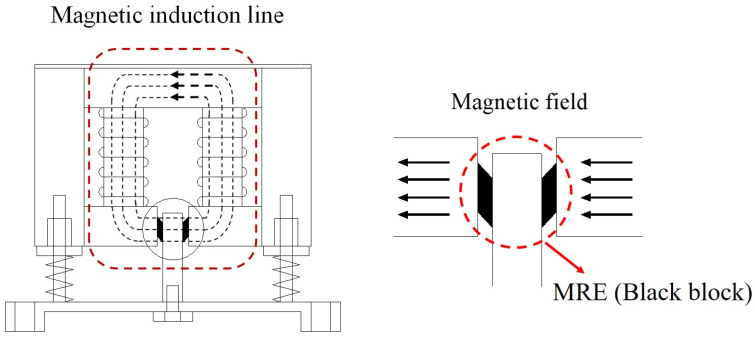
Construction principle of frequency adjustable tuned mass damper (FATMD). The parts in the dotted lines indicate the position of magnetic induction line and magnetorheological elastomer (MRE) respectively.

The basic structure of FATMD is shown in Figure 2. The working mechanism of FATMD is described as follows.

When the natural frequency of the main structure changes, the magnetic field in the magnetic circuit is changed by inputting a corresponding magnitude of current to the FATMD coil, thereby adjusting the shear modulus of MRE and the frequency of FATMD. Compared with traditional TMD, FATMD can maintain the optimal tuning state with the main structure in real time, and overcome the frequency sensitivity problem of traditional TMD.

### 2.3. Magnetic Circuit Analysis of FATMD

**Figure 3:**
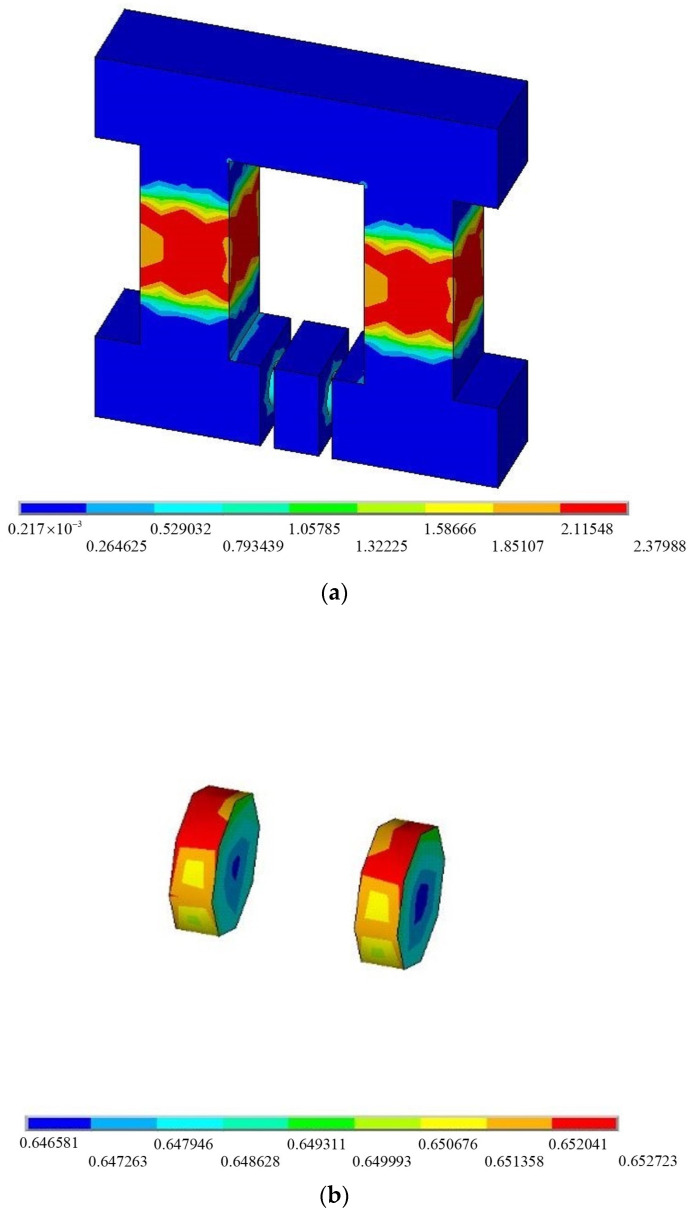
Magnetic induction distribution of FATMD: (**a**) Global model; (**b**) Magnetorheological elastomer (MRE).

The main components of the selected MRE are 15% rubber, 15% silicone oil and 70% hydroxy iron powder [50]. Among them, rubber is used as the base material, silicone oil is used to mix iron powder into liquid rubber, and hydroxyl iron powder is used for magnetic particles, due to its high magnetism efficiency at a high frequency [51]. We used ANSYS to complete the FATMD three-dimensional modelling and magnetic circuit analysis. Figure 3 shows the ANSYS magnetic circuit analysis cloud diagram under 2 A current. The magnetic induction of the iron core of the magnetic circuit is the largest, followed by the MRE, and the magnetic induction distribution in the MRE region is relatively uniform, with an average value of 0.647 T.

**Figure 4:**
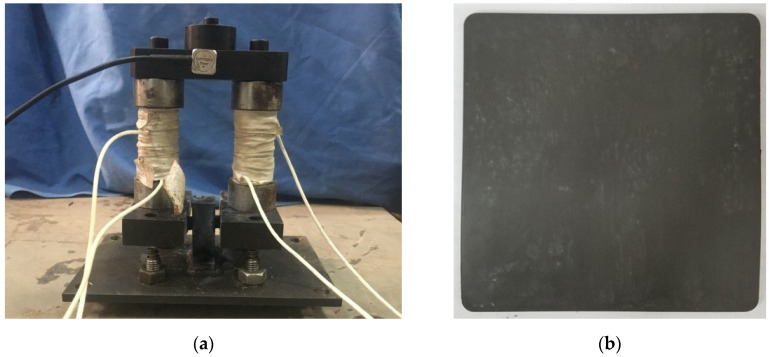
Real objects of FATMD: (**a**) FATMD; (**b**) MRE.

**Figure 5:**
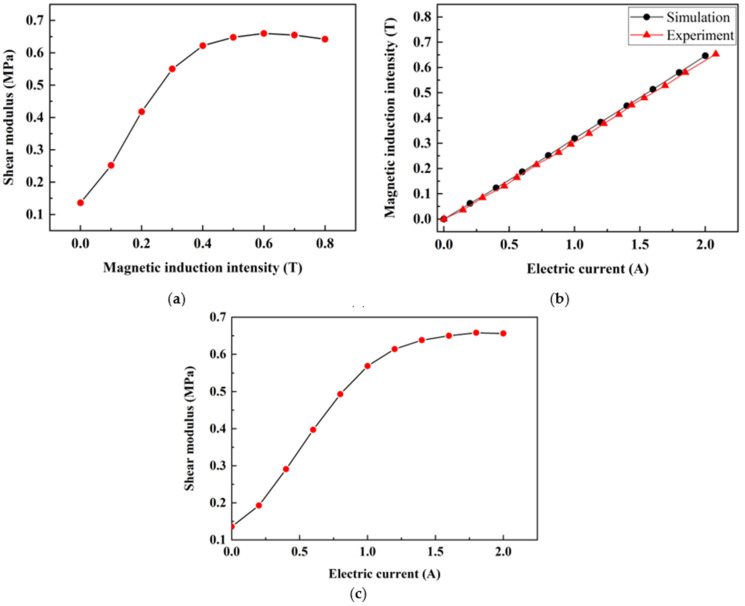
Relationship between material properties in MRE: (**a**) Magnetic induction and shear modulus of MRE; (**b**) Current and magnetic induction; (**c**) Current and shear modulus.

Based on the above analysis, we made FATMD, and a photo of the real object is shown in Figure 4. MRE is pasted between the lower bottom plate and the upper magnetic conductor to facilitate shear deformation. The mass of FATMD is provided by the panel, additional weight, iron core and magnetic conductor, and its stiffness is provided by the MRE and spring. Variable current can be input into the coil to change the stiffness of FATMD. Under different magnetic induction, the shear modulus of MRE can be measured by dynamic mechanical analyzer (DMA), as shown in Figure 5a. We measured the magnetic induction at the MRE in the magnetic circuit with a Gauss meter, and compared it with the ANSYS magnetic circuit analysis result, as shown in Figure 5b. It can be seen that in the range of 0–2 A, the MRE magnetic induction has a linear relationship with the current, and the measured data is in good agreement with the simulation results, which shows that the FATMD magnetic circuit design is successful. Combining the measured data in Figure 5a,b, the relationship between the input current and the MRE shear modulus can be fitted, as shown in Figure 5c. It can be seen that the shear modulus of MRE increases with the increase in the input current. When the current is between 0 and 1A, the shear modulus of MRE increases approximately linearly with the current. When the current is greater than 1 A, the growth rate of the modulus gradually slows down with the increase in current and, finally, the MRE shear modulus reaches the maximum value when the current reaches about 1.8 A.

## 3. Real-Time Identification of System Natural Frequency

Due to the fact that the fast Fourier transform (FFT) can only obtain the frequency domain characteristics of the selected signal interval [52], and it is easy to cause TMD detuning [53], we used the HHT and NExT methods to identify the natural frequency of the system. As such, HHT is composed of the following two parts: empirical mode decomposition (EMD) and Hilbert transform (HT). EMD decomposes the signal into intrinsic mode function (IMF), based on the time-scale characteristics of the data and NExT uses the cross-correlation function between the two points of the structure under stationary random vibration signal excitation, to replace the impulse response function [54], so that the real-time natural frequency of the system can be determined.

### 3.1. Hilbert–Huang Transformation

In order to make the signal meet the requirements of the HT, the IMFs need to be screened. The method [55] is as follows:

First, the upper and lower envelopes of the signal need to be averaged, and the remainder term is obtained by continuously removing the envelope average.

(6) $$\left\{\begin{array}{l} h_{i+1,j+1}\left(t\right)=h_{i+1,j}\left(t\right)-m_{i+1,j}\left(t\right)\left(i=0,1,2,\cdots ,j=0,1,2,\cdots \right)\\ x\left(t\right)=h_{1,0}\left(t\right) \end{array}\right.$$

where $h_{i+1,j+1}\left(t\right)$ represents the i+1 residual term after the j+1 screening; $m_{i+1,j}\left(t\right)$ is the average value of $h_{i+1,j}\left(t\right)$; $x\left(t\right)$ is the initial input signal.

The remainder term $h_{i+1,j+1}\left(t\right)$ is required to meet the IMF restrictions [49], which is defined as follows:

(7) $$SD_{i,j+1}=\sum_{t=0}^{t}\left[\frac{{\left|h_{i+1,j}\left(t\right)-h_{i+1,j+1}\left(t\right)\right|}^{2}}{h_{i+1,j}^{2}}\right]$$

(8) $$x\left(t\right)=\sum_{i=0}^{n}c_{i+1}\left(t\right)+r$$

where the smaller the value of $SD_{i,j+1}$, the better the stability of the IMF, and the more conducive to the HT. If the value of $SD_{i,j+1}$ is between 0.2 and 0.3, the screening will be stopped; otherwise, the screening will continue until all the conditions are met, and it will be regarded as the IMF $c_{i+1}\left(t\right)$. Repeat the work above until the r in Equation (8) becomes a monotonic function and cannot meet the IMF.

Then, perform HT on the IMF of each order, assuming a set of given signals is $y\left(t\right)$, and the HT formula can be written as follows:

(9) $$\hat{y}\left(t\right)=H\left[y\left(t\right)\right]=\frac{1}{\pi }\int_{-\infty }^{\infty }\frac{y\left(\tau \right)}{t-\tau }d\tau$$

The analytic signal formed is as follows:

(10) $$Y\left(t\right)=y\left(t\right)+i\hat{y}\left(t\right)=A\left(t\right)e^{i\theta \left(t\right)}$$

(11) $$A\left(t\right)=\sqrt{y^{2}\left(t\right)+{\hat{y}}^{2}\left(t\right)},\theta \left(t\right)=\arctan2\left(\frac{\hat{y}\left(t\right)}{y\left(t\right)}\right)$$

where $A\left(t\right)$ is the instantaneous amplitude; $\theta \left(t\right)$ is the instantaneous phase.

Thus, the instantaneous frequency can be obtained, as follows:

(12) $$\omega \left(t\right)=\frac{d\theta \left(t\right)}{dt}$$

### 3.2. Natural Excitation Technique

The IMF obtained by EMD is a stable random signal, which is suitable for NExT to calculate its free attenuation response. Assuming that the degree of freedom of the system is N, the measurement point and the reference point are set to $q_{1}$ and $q_{2}$, respectively. When a point *p* of the structure is excited by white noise, the cross-correlation function between $q_{1}$ and $q_{2}$ is as follows:

(13) $$\begin{array}{l} R_{q_{1}q_{2},p}\left(T\right)=E\left(z_{q_{1}}\left(t+T\right)z_{q_{2}}\left(t\right)\right)\\ \;\;\;\;\;\;\;\;\;\;\;\;\;\;=\sum_{l_{1}=1}^{N}\sum_{l_{2}=1}^{N}\varphi_{l_{1},q_{1}}\varphi_{l_{1},p}\varphi_{l_{2},q_{2}}\varphi_{l_{2},p}E\left(Z_{l_{1},l_{2}}\right) \end{array}$$

(14) $$Z_{l_{1},l_{2}}=\int_{-\infty }^{t+T}\int_{-\infty }^{t}\frac{d\left(\tau_{1}\right)}{m_{l_{1}}\omega_{d,l_{1}}}e^{-\xi_{l_{1}}\omega_{l_{1}}\left(t+T-\tau_{1}\right)}\sin\left(\omega_{d,l_{1}}\left(t+T-\tau_{1}\right)\right)\frac{d\left(\tau_{2}\right)}{m_{l_{2}}\omega_{d,l_{2}}}e^{-\xi_{l_{2}}\omega_{l_{2}}\left(t-\tau_{2}\right)}\sin\left(\omega_{d,l_{2}}\left(t-\tau_{2}\right)\right)d\tau_{1}d\tau_{2}$$

(15) $$\omega_{d,l_{1}}=\omega_{l_{1}}{\left(1-\xi_{l_{1}}^{2}\right)}^{0.5},\omega_{d,l_{1}}=\omega_{l_{1}}{\left(1-\xi_{l_{1}}^{2}\right)}^{0.5}$$

where $l_{1}$ and $l_{2}$ are the modal orders, corresponding to point $q_{1}$ and point $q_{2}$, respectively; $\varphi_{l_{n},q_{n}}$ and $\varphi_{l_{n},p}$ are the responses of point $q_{n}$ and point p under the $l_{n}$th mode shape $\left(n=1,2\right)$; $m_{l_{n}}$ is the $l_{n}$-th modal mass; $\omega_{l_{n}}$ is the $l_{n}$-th natural frequency; $\xi_{l_{n}}$ is the $l_{n}$-th modal damping ratio; d is white noise excitation.

The cross-correlation function of white noise can be expressed as follows:

(16) $$E\left(d\left(\tau_{1}\right)d\left(\tau_{2}\right)\right)=\alpha_{p}\delta \left(\tau_{1}-\tau_{2}\right)$$

where $\alpha_{p}$ is the constant related to excitation; δ is Dirac function.

Substituting Equation (16) into Equation (13), we can obtain the following:

(17) $$\begin{array}{l} R_{q_{1}q_{2},p}\left(T\right)\;=\sum_{l_{1}=1}^{N}\sum_{l_{2}=1}^{N}\alpha_{p}\varphi_{l_{1},q_{1}}\varphi_{l_{1},p}\varphi_{l_{2},q_{2}}\varphi_{l_{2},p}Z_{2}\\ \;\;\;\;\;\;\;\;\;\;\;\;\;\;\;\;=\sum_{l_{1}=1}^{N}\frac{\varphi_{l_{1},q_{1}}A_{q_{2}}^{l_{1}}}{m_{l_{1}}\omega_{d,l_{1}}}e^{-\xi_{l_{1}}\omega_{l_{1}}T}\sin\left(\omega_{d,l_{1}}T+\Theta_{l_{1}}\right) \end{array}$$

(18) $$Z_{2}=\int_{-\infty }^{t}\frac{\sin\left(\omega_{d,l_{1}}\left(t+T-\tau_{2}\right)\right)}{m_{l_{1}}\omega_{d,l_{1}}}e^{-\xi_{l_{1}}\omega_{l_{1}}\left(t+T-\tau_{2}\right)}\frac{\sin\left(\omega_{d,l_{2}}\left(t-\tau_{2}\right)\right)}{m_{l_{2}}\omega_{d,l_{2}}}e^{-\xi_{l_{2}}\omega_{l_{2}}\left(t-\tau_{2}\right)}d\tau_{2}$$

where $A_{q_{2}}^{l_{1}}$ and $\Theta_{l_{1}}$ are the constant terms related to reference point $q_{2}$ and modal order $l_{1}$. It can be seen from Equations (17) and (18) that under the excitation of white noise, the cross-correlation function of the two points is equivalent to the accumulation of a series of impulse response signals, which can be used to replace impulse response signals.

### 3.3. The Procedures of the System Natural Frequency Identification Method

The HHT+NExT method divides HHT into two parts, i.e., EMD and HT. The main implementation process is as follows:

- Firstly, obtain the natural frequency of the system response signal under impulse excitation by FFT;
- According to the natural frequency of the mode to be controlled, set a band-pass filter that allows the natural frequency signal to pass through to reduce the influence of noise on the response signal and reduce the modal aliasing phenomenon in HHT;
- Set the measurement point and reference point, delay the response signal to obtain the filtered measurement point signal array and the reference point signal array and use EMD for the two sets of signals to obtain the corresponding *i*th IMF signals;
- To eliminate the end effect of the signal array of measurement point and reference point, we use NExT to process them, and obtain the impulse response signal corresponding to the *i*th mode, and perform HT to obtain the mean value of the instantaneous frequency corresponding to the *i*th mode;
- The natural frequency of the system will be updated based on the average value of the output instantaneous frequency at a certain pre-set time interval.

To verify the effectiveness of the real-time frequency identification method, we carried out a case study on a fixed-supported steel beam structure to identify the first three natural frequencies of the steel beam system, and compared them with the ANSYS finite element analysis results. The parameters of the steel beam are shown in Table 1.

**Figure 6:**
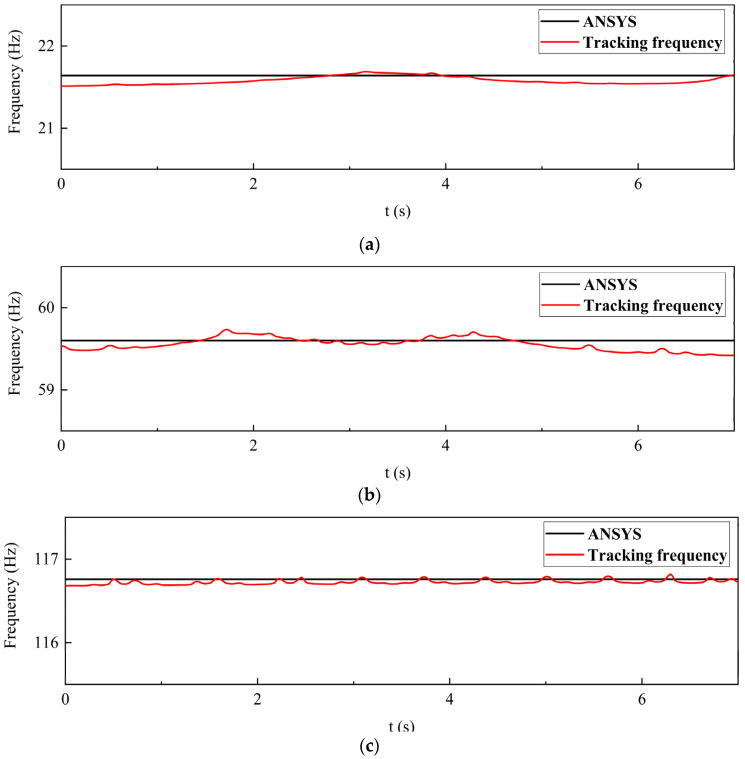
Comparison of identification frequency and modal analysis frequency of ANSYS finite element software: (**a**) First-order natural frequency; (**b**) Second-order natural frequency; (**c**) Third-order natural frequency.

Through ANSYS finite element analysis, the first three natural frequencies of the steel beam structure were 21.64 Hz, 59.60 Hz, and 116.76 Hz. The frequency identification method introduced in Section 3.3 was used to intercept the acceleration response signal within 7 s, to identify the natural frequency of the system. Figure 6 shows the comparison result of the identification frequency and the frequency obtained from ANSYS analysis, which indicates a good agreement.

## 4. FATMD Real-Time Tracking Method of the Natural Frequency of the Main Structure

On the basis of the real-time identification of the natural frequency of the system, we also need to calculate the natural frequency of the main structure through the TMD optimal design theory, so that the natural frequency of FATMD can track the natural frequency of the main structure in real time, in order to achieve the optimal tuning state.

The main structure and FATMD are equivalent to a two-degree-of-freedom system. The first two natural frequencies of the FATMD-main structure vibration reduction system are obtained by identifying the vibration response signal, and then the natural frequency of the main structure is calculated according to the first two natural frequencies and the formula of the acceleration amplitude ratio.

The calculation method is as follows:

- Firstly, the derivative of the acceleration amplitude ratio formula, with respect to the square of the external excitation frequency ratio, is calculated to obtain the modal mass expression of the main structure when the derivative is 0;
- Then, the first two natural frequencies of the identified system are substituted into the above expression to obtain two modal mass expressions of the main structure with different coefficients;
- Finally, under the condition that the stiffness, mass and natural frequency of FATMD are known, the above two expressions are combined to obtain the modal stiffness of the main structure, and then the modal mass of the main structure and the optimal design parameters of FATMD are obtained.

According to the above method, it can be obtained from Equation (4), as follows:

(19) $$\frac{d}{d\left(\lambda^{2}\right)}\left(\frac{\lambda^{4}\left({\left(\gamma^{2}-\lambda^{2}\right)}^{2}+{\left(2\xi \lambda \right)}^{2}\right)}{\left({\left(\left(1-\lambda^{2}\right)\left(\gamma^{2}-\lambda^{2}\right)-\mu \gamma^{2}\lambda^{2}\right)}^{2}+{\left(1-\left(1+\mu \right)\lambda^{2}\right)}^{2}{\left(2\xi \lambda \right)}^{2}\right)}\right)=0$$

Substituting Equation (5) into Equation (19), the modal mass of the main structure can be obtained as:

(20) $$m_{1}^{\prime }=\frac{M_{11}+M_{12}}{M_{13}}=\frac{M_{21}+M_{22}}{M_{23}}$$

(21) $$\begin{array}{l} M_{11}=2c_{2}^{4}k_{1}\omega_{f1}^{4}\left(k_{1}-m_{2}\omega_{f1}^{2}\right)+c_{2}^{2}m_{2}^{2}\omega_{f1}^{2}\left(\begin{array}{l} m_{2}^{2}\omega_{f1}^{8}+4k_{1}^{2}{\left(\omega_{f1}^{2}-\omega_{2}^{2}\right)}^{2}-\\ 2k_{1}m_{2}\omega_{f1}^{2}\left(2\omega_{f1}^{4}-3\omega_{f1}^{2}\omega_{2}^{2}+2\omega_{2}^{4}\right) \end{array}\right)\\ M_{12}=2m_{2}^{4}\left(\omega_{f1}^{2}-\omega_{2}^{2}\right)\left(\begin{array}{l} m_{2}^{2}\omega_{f1}^{6}\omega_{2}^{4}+k_{1}^{2}{\left(\omega_{f1}^{2}-\omega_{2}^{2}\right)}^{3}+\\ k_{1}m_{2}\omega_{f}^{2}\omega_{2}^{2}\left(2\omega_{f1}^{4}-3\omega_{f1}^{2}\omega_{2}^{2}+\omega_{2}^{4}\right) \end{array}\right)\\ M_{13}=2\omega_{f1}^{2}\left(\begin{array}{l} c_{2}^{4}k_{1}\omega_{f1}^{4}+m_{2}^{4}{\left(\omega_{f1}^{2}-\omega_{2}^{2}\right)}^{2}\left(m_{2}\omega_{f1}^{4}\omega_{2}^{2}+k_{1}{\left(\omega_{f1}^{2}-\omega_{2}^{2}\right)}^{2}\right)+\\ c_{2}^{2}m_{2}^{2}\omega_{f1}^{2}\left(-m_{2}\omega_{f1}^{6}+2k_{1}{\left(\omega_{f1}^{2}-\omega_{2}^{2}\right)}^{2}\right) \end{array}\right) \end{array}$$

(22) $$\begin{array}{l} M_{21}=2c_{2}^{4}k_{1}\omega_{f2}^{4}\left(k_{1}-m_{2}\omega_{f2}^{2}\right)+c_{2}^{2}m_{2}^{2}\omega_{f2}^{2}\left(\begin{array}{l} m_{2}^{2}\omega_{f2}^{8}+4k_{1}^{2}{\left(\omega_{f2}^{2}-\omega_{2}^{2}\right)}^{2}-\\ 2k_{1}m_{2}\omega_{f2}^{2}\left(2\omega_{f2}^{4}-3\omega_{f2}^{2}\omega_{2}^{2}+2\omega_{2}^{4}\right) \end{array}\right)\\ M_{22}=2m_{2}^{4}\left(\omega_{f2}^{2}-\omega_{2}^{2}\right)\left(\begin{array}{l} m_{2}^{2}\omega_{f2}^{6}\omega_{2}^{4}+k_{1}^{2}{\left(\omega_{f2}^{2}-\omega_{2}^{2}\right)}^{3}+\\ k_{1}m_{2}\omega_{f2}^{2}\omega_{2}^{2}\left(2\omega_{f2}^{4}-3\omega_{f2}^{2}\omega_{2}^{2}+\omega_{2}^{4}\right) \end{array}\right)\\ M_{23}=2\omega_{f2}^{2}\left(\begin{array}{l} c_{2}^{4}k_{1}\omega_{f2}^{4}+m_{2}^{4}{\left(\omega_{f2}^{2}-\omega_{2}^{2}\right)}^{2}\left(m_{2}\omega_{f2}^{4}\omega_{2}^{2}+k_{1}{\left(\omega_{f2}^{2}-\omega_{2}^{2}\right)}^{2}\right)+\\ c_{2}^{2}m_{2}^{2}\omega_{f2}^{2}\left(-m_{2}\omega_{f2}^{6}+2k_{1}{\left(\omega_{f2}^{2}-\omega_{2}^{2}\right)}^{2}\right) \end{array}\right) \end{array}$$

where $\omega_{f1}$ and $\omega_{f2}$ are the first and second natural frequencies of the FATMD-main structure vibration reduction system; $k_{1}$ is the modal stiffness of the main structure; $m_{1}^{\prime }$ is the modal mass of the main structure.

The $\left(M_{11}+M_{12}\right)/M_{13}$ and $\left(M_{21}+M_{22}\right)/M_{23}$ in Equation (20) represent the modal mass of the main structure. When the first two natural frequencies of the system and the mass, stiffness and natural frequency of FATMD are known, the $\left(M_{11}+M_{12}\right)/M_{13}$ and $\left(M_{21}+M_{22}\right)/M_{23}$ can be combined to calculate the modal stiffness $k_{1}$ of the main structure, and the modal mass $m_{1}^{\prime }$ can be obtained by substituting Equation (20).

Then, the first-order natural frequency of the main structure can be obtained, as follows:

(23) $$f_{1}=\frac{1}{2\pi }\sqrt{\frac{k_{1}}{m_{1}^{\prime }}}$$

Combining Equation (3), the optimal frequency of FATMD can be obtained, as follows:

(24) $$f_{2}=f_{1}\sqrt{\frac{m_{1}^{\prime }}{m_{1}^{\prime }+m_{2}}}$$

The optimal stiffness of FATMD is as follows:

(25) $$k_{2}=4\pi^{2}f_{2}^{2}m_{2}$$

For the self-made FATMD in this study, its stiffness can be expressed as follows:

(26) $$\begin{array}{l} k_{2}=4k+2k_{m}\\ k_{m}=\frac{G_{m}A}{h} \end{array}$$

where k is the spring stiffness; $k_{m}$, $G_{m}$, A, h are the MRE stiffness, shear modulus, area and thickness, respectively. It can be seen from Figure 5b that $G_{m}$ in Equation (26) can be adjusted by the current in the FATMD coil, thereby changing the stiffness and natural frequency of the FATMD, and realizing the real-time tracking of the natural frequency of the FATMD to the natural frequency of the main structure.

## 5. Vibration Control Experiment of FATMD

**Figure 7:**
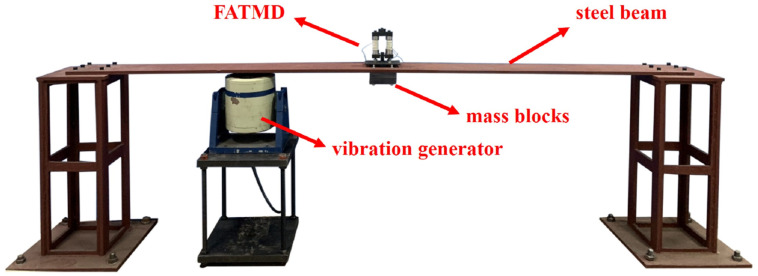
Vibration control experiment platform.

To verify the vibration reduction effect of FATMD, we built a vibration control experiment platform, as shown in Figure 7. In the experiment, the natural frequency of the steel beam is adjusted by changing the number of mass blocks in the middle of the steel beam, which is used to simulate the change of the natural frequency of the main structure. FATMD is placed in the middle of the steel beam to control the first-order modal vibration of the structure.

There are five comparative experiment cases, including four additional mass blocks, three mass blocks, two mass blocks, one mass block and no mass blocks. Through frequency identification, the first-order natural frequencies of the five experiment cases are 19.41 Hz (19.42 Hz), 19.91 Hz (19.96 Hz), 20.44 Hz (20.53 Hz), 21.01 Hz (21.11 Hz) and 21.64 Hz (21.71 Hz), the recognition errors are within 1%. It can be seen that the identified natural frequency is accurate, which confirms that the proposed real-time frequency tracking method is applicable and can be used to calculate the natural frequency of the main structure.

**Figure 8:**
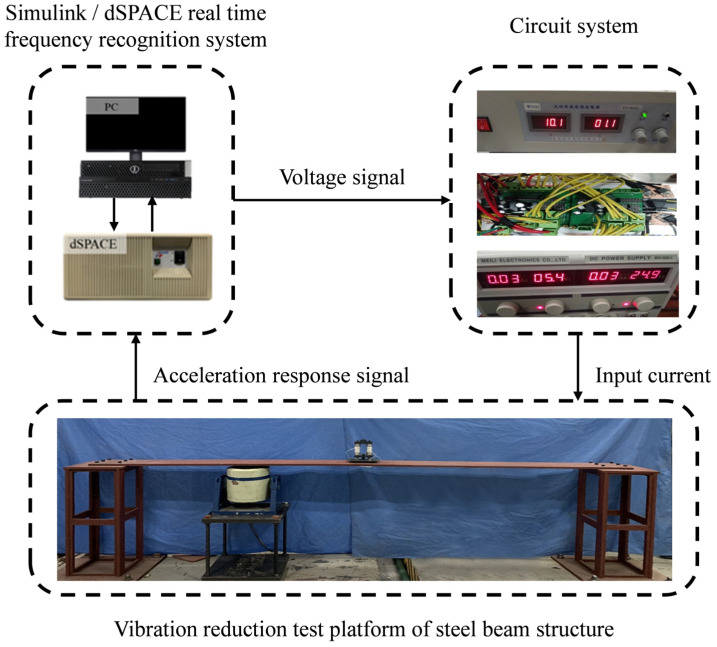
FATMD vibration reduction experiment system.

The parameters of FATMD are shown in Table 2, in which MRE is a cylindrical block, with a cross-sectional area of 33 mm^2^ and a thickness of 3 mm. The working process of the FATMD vibration reduction experiment system is shown in Figure 8. The frequency tracking system in the experiment consists of Simulink/dSPACE and a circuit system. The circuit system is composed of a high-voltage source and a low-voltage source, respectively, connected to the FATMD coil and the dSPACE output. The principle is as follows.

Firstly, the real-time frequency tracking method, proposed in Section 3.3, is realized by Simulink/dSPACE, and the first two natural frequencies of the system are obtained. Then, the natural frequencies of the main structure are calculated by Equations (20) and (23), which are substituted into Equations (24)–(26) to calculate the optimal frequency and stiffness of FATMD. Combined with the relationship between the current and the shear modulus in Figure 5c and the relationship between voltage and current in the circuit system, the current in the FATMD coil is adjusted to make the FATMD achieve the optimal tuning state. 

**Figure 9:**
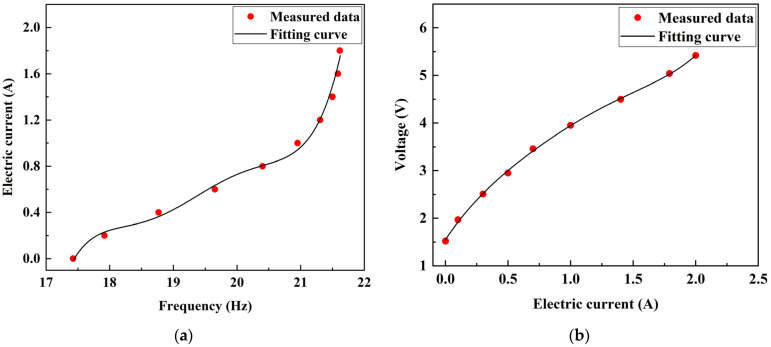
Relationship between physical quantities of circuit system: (**a**) Natural frequency of FATMD; (**b**) Input current and between input current and voltage.

We obtained the function relationship between frequency and current, current and voltage, through the measured data of current and voltage in the circuit system, as shown in Figure 9. The relationship between input voltage and FATMD natural frequency is shown in Equation (27).

(27) $$U=1.548+4.020I-3.344I^{2}+2.916I^{3}-1.504I^{4}+0.311I^{5}$$

where

(28) $$I=-73702.868+19132.147f_{2}-19844.901f_{2}^{2}+102.812f_{2}^{3}-2.660f_{2}^{4}+0.028f_{2}^{5}$$

To verify the vibration reduction effect of FATMD under the changes in stiffness of the main structure, we conducted vibration control experiments using traditional TMD and FATMD. The mass of the traditional TMD is 2.25 kg; the total spring stiffness is 32,636 N/m, and the damping ratio is 0.09. The specific experiment steps are as follows:

- First, taking the four mass blocks in the middle of the steel beam as the basic case, conduct the steel beam vibration experiment in an uncontrolled state, and record the mid-span acceleration response of the steel beam under harmonic and white noise excitation;
- Place the traditional TMD in the middle of the steel beam and record the mid-span acceleration response;
- TMD is replaced by FATMD. Apply voltage to FATMD through the calculation frequency of the main structure, using Equation (27). Then, record the mid-span acceleration response under the two excitation methods;
- Reduce the number of mass blocks to achieve the purpose of changing the natural frequency of the steel beam, repeat the above steps until the number of mass blocks is zero.

**Figure 10:**
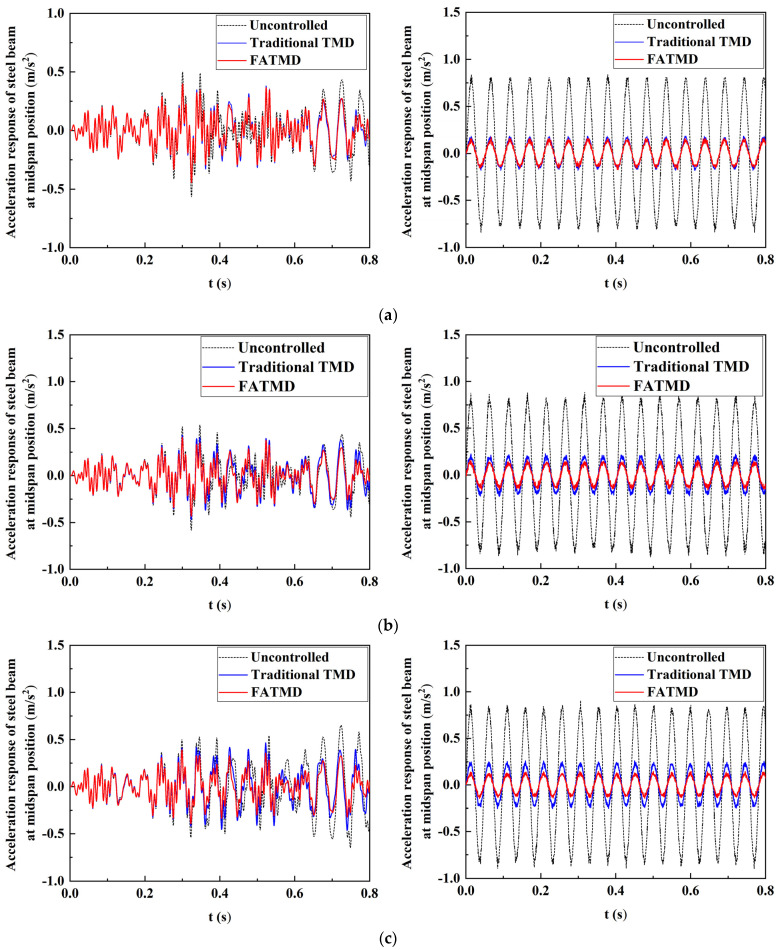

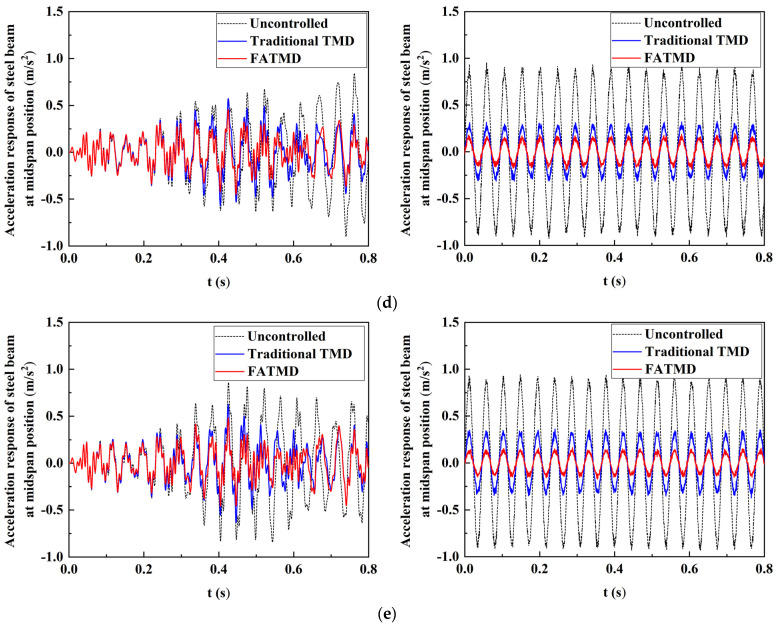
Comparison of acceleration response in the mid-span of steel beam: (**a**) 4 mass blocks; (**b**) 3 mass blocks; (**c**) 2 mass blocks; (**d**) 1 mass block; (**e**) 0 mass blocks.

The acceleration response curves of the steel beam under different cases are as shown in Figure 10. In each subfigure, the case of white noise excitation is on the left and that of natural frequency excitation is on the right. It can be seen that in the steady-state response stage of the system, when the external excitation is white noise, the acceleration of the FATMD vibration reduction system remains at about 0.4 m/s^2^ with the gradual decrease in the number of mass blocks. The minimum and maximum vibration reduction ratios of peak acceleration are 21.83% and 49.73%, respectively, which are significantly better than those of the traditional TMD vibration reduction system. Similarly, when the external excitation is harmonic excitation, and the peak acceleration of the FATMD vibration reduction system is always maintained at about 0.1 m/s^2^ with the reduction in the number of mass blocks. However, the peak acceleration of the traditional TMD vibration reduction system is increased from 0.19 m/s^2^ to 0.35 m/s^2^. It indicates that FATMD can adapt to the frequency changes of the main structure and maintain the optimum vibration reduction effect.

**Figure 11:**
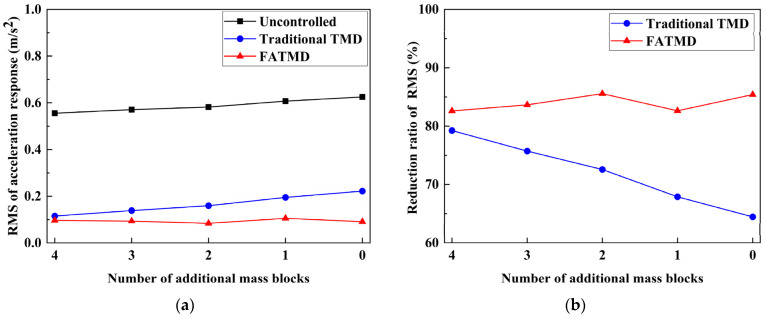
RMS and its reduction in acceleration response at mid-span of steel beam with different numbers of additional mass blocks under harmonic excitation: (**a**) RMS; (**b**) the reduction ratio of RMS.

In order to show more clearly that the traditional TMD is more sensitive to the change of the natural frequency of the main structure and highlight the advantages of FATMD, we calculated the RMS and its reduction of the mid-span acceleration response of the steel beam without control, with TMD and with FATMD, under harmonic excitation, as shown in Figure 11. It was found that under harmonic excitation, as the number of additional mass blocks decreases, the acceleration response, RMS of FATMD, is basically maintained at about 0.1 m/s^2^, and the vibration reduction ratio is between 82.61% and 85.56%, which appears to be steady. The trend is due to the frequency tracking method, which can be used to observe the natural frequency of the main structure in real time, so as to ensure that FATMD is always in the optimal tuning state. The acceleration response, RMS, of the traditional TMD increased from 0.12 m/s^2^ to 0.22 m/s^2^, and the vibration reduction ratio decreased from 79.24% to 64.45%. The vibration reduction ratio continues to decrease, which indicates that the natural frequency of the steel beam changes when the number of mass blocks is reduced. That is, when the natural frequency of the main structure changes, the traditional TMD appears to be detuning. Therefore, FATMD can obtain a stable and good vibration reduction effect in a wide frequency band, and its vibration reduction performance is significantly better than that of traditional TMD.

## 6. Conclusions

We developed a FATMD, based on the smart material MRE, and a frequency tracking system based on Simulink/dSPACE. We also carried out the vibration control experiments of FATMD on fixed-supported steel beams. The following main conclusions are drawn from this study:

- FATMD can change the natural vibration frequency by changing the input current, so as to adapt to the frequency change of the main structure, and continuously achieve an optimal vibration reduction effect. Based on the FATMD magnetic circuit analysis and MRE magnetic induction tests, the relationship between the MRE shear modulus and input current is obtained, and then a method for adjusting the natural frequency of FATMD is proposed.
- A real-time frequency tracking method, based on HHT+NExT, is established. The simulation and experimental results of frequency tracking show that the method has high accuracy for frequency identification of the main structure, which confirms the applicability of the proposed method. The identified natural frequency of the vibration reduction system can be converted into the natural frequency of the main structure through the TMD optimal design theory, and the optimal frequency of FATMD can be calculated using the natural frequency of the main structure. Based on the relationship between the optimal frequency and the voltage of FATMD, the frequency tracking of FATMD can be achieved.
- It was found that, compared with traditional TMD, FATMD has better and stable vibration reduction effect when the natural frequency of the main structure changes. When the difference between the natural frequency of the structure and the natural frequency of the TMD gradually increases, the traditional TMD appears to be detuning, and the vibration reduction effect is reduced. However, FATMD can track the natural frequency of the structure in real time to continue to maintain good vibration reduction performance.

## Figures and Tables

**Table 1:** Parameters of steel beam structure.

Size (m^3^)	Elastic Modulus (GPa)	Poisson’s Ratio	Damping Ratio	Density (kg/m^3^)
2.5 × 0.24 × 0.025	211	0.3	0.01	7800

**Table 2:** Basic performance parameters of FATMD.

Mass (kg)	Damping Ratio	Current (A)	Stiffness without Current (N/m)	Max Stiffness (N/m)	MRE Size (mm^3^)
2.25	0.09	0~2	26,956	41,519	33 × 3

## Data Availability

The data presented in this study are available on request from the corresponding author.

## References

[1] Chang C., Shia S., Lai Y. (2018). Seismic design of passive tuned mass damper parameters using active control algorithm. J. Sound Vib..

[2] Elias S., Matsagar V. (2017). Research developments in vibration control of structures using passive tuned mass dampers. Annu. Rev. Control.

[3] Meinhardt C., Newland D., Talbot J., Taylor D. Vibration performance of London’s Millennium Footbridge. Proceedings of the 24th International Congress on Sound and Vibration (ICSV24).

[4] Wang D., Wu C., Zhang Y., Li S. (2019). Study on vertical vibration control of long-span steel footbridge with tuned mass dampers under pedestrian excitation. J. Constr. Steel Res..

[5] Ferreira F., Simões L. (2019). Optimum Design of a Controlled Cable-Stayed Footbridge Subject to a Running Event Using Semiactive and Passive Mass Dampers. J. Perform. Constr. Facil..

[6] Bathaei A., Zahrai S.M., Ramezani M. (2018). Semi-active seismic control of an 11-DOF building model with TMD+MR damper using type-1 and -2 fuzzy algorithms. J. Vib. Control.

[7] Chen Z., Fang H., Han Z., Sun S. (2019). Influence of bridge-based designed TMD on running trains. J. Vib. Control.

[8] Yang F., Sedaghati R., Esmailzadeh E. (2021). Vibration suppression of structures using tuned mass damper technology: A state-of-the-art review. J. Vib. Control.

[9] Rana R., Soong T.T. (1998). Parametric study and simplified design of tuned mass dampers. Eng. Struct..

[10] Wang J.F., Lin C.C., Chen B.L. (2003). Vibration suppression for high-speed railway bridges using tuned mass dampers. Int. J. Solids Struct..

[11] Okhovat M.R., Rahimian M., Ghorbani-Tanha A.K. Tuned mass damper for seismic response reduction of Tehran Tower. Proceedings of the 4th International Conference on Earthquake Engineering.

[12] Wen Y., Sun L. (2011). Research on wind response control of large cable-stayed bridge under construction by using hybrid system of TMDs and ATMDs. Eng. Mech..

[13] Jaiswal O.R. Simple tuned mass damper to control seismic response of elevated tanks. Proceedings of the 13th World Conference on Earthquake Engineering.

[14] Suzuki S., Fujino E., Noguchi H. (2004). Experimental study on effect of human-load on vertical dynamic characteristics of wooden floor (part 1). J. Struct. Constr. Eng..

[15] Oyguc R., Toros C., Abdelnaby A.E. (2018). Seismic behavior of irregular reinforced-concrete structures under multiple earthquake excitations. Soil. Dyn. Earthq. Eng..

[16] Mantawy A., Anderson J.C. (2018). Effect of long-duration earthquakes on the low-cycle fatigue damage in RC frame buildings. Soil Dyn. Earthq. Eng..

[17] Koto Y., Konishi T., Sekiya H., Miki C. (2019). Monitoring local damage due to fatigue in plate girder bridge. J. Sound Vib..

[18] Minaei A., Ghorbani-Tanha A.K. (2019). Optimal step-by-step tuning method for variable stiffness semiactive tuned mass dampers. J. Eng. Mech..

[19] Wang Z., Gao H., Wang H., Chen Z. (2019). Development of stiffness-adjustable tuned mass dampers for frequency retuning. Adv. Struct. Eng..

[20] Karami K., Manie S., Ghafouri K., Nagarajaiah S. (2019). Nonlinear structural control using integrated DDA/ISMP and semi-active tuned mass damper. Eng. Struct..

[21] Berardengo M., Cigada A., Guanziroli F., Manzoni S. An adaptive tuned mass damper based on shape memory alloys with an extended range of frequency. Proceedings of the 2014 IEEE Workshop on Environmental, Energy, and Structural Monitoring Systems Proceedings.

[22] Lin G.L., Lin C.C., Chen B.C., Soong T.T. (2015). Vibration control performance of tuned mass dampers with resettable variable stiffness. Eng. Struct..

[23] Huo L., Huang C., Zhang Y., Li H. (2021). A passive adaptive suspended mass pendulum to compensate detuning due to large swing angle. Int. J. Struct. Stab. Dy..

[24] Tuan A., Shang G.Q. (2014). Vibration control in a 101-storey building using a tuned mass damper. J. Appl. Sci. Eng..

[25] Lu X., Zhang Q., Weng D., Zhou Z., Wang S., Mahin S.A., Ding S., Qian F. (2017). Improving performance of a super tall building using a new eddy-current tuned mass damper. Struct. Control Health Monit..

[26] Sun C., Nagarajaiah S. (2014). Study on semi-active tuned mass damper with variable damping and stiffness under seismic excitations. Struct. Control Health Monit..

[27] Nagarajaiah S. (2010). Adaptive Passive, Semiactive, Smart Tuned Mass Dampers: Identification and Control Using Empirical Mode Decomposition, Hilbert Transform, and Short-Term Fourier Transform. Struct. Control Health Monit..

[28] Shi W., Wang L., Lu Z. (2018). Study on self-adjustable tuned mass damper with variable mass. Struct. Control Health Monit..

[29] Shi W., Wang L., Lu Z., Wang H. (2019). Experimental and numerical study on adaptive-passive variable mass tuned mass damper. J. Sound Vib..

[30] Samal S. (2020). Effect of shape and size of filler particle on the aggregation and sedimentation behavior of the polymer composite. Powder Technol..

[31] Samal S., Blanco I. (2021). Investigation of Dispersion, Interfacial Adhesion of Isotropic and Anisotropic Filler in Polymer Composite. Appl. Sci..

[32] Samal S., Škodová M., Abate L., Blanco I. (2020). Magneto-Rheological Elastomer Composites. A Review. Appl. Sci..

[33] Jaafar M.F., Mustapha F., Mustapha M. (2021). Review of current research progress related to magnetorheological elastomer material. J. Mater. Res. Technol..

[34] Jeong U.C., Yoon J.H., Yang I.H., Järvinen E., Kärnä T. (2013). Magnetorheological elastomer with stiffness-variable characteristics based on induced current applied to differential mount of vehicles. Smart Mater. Struct..

[35] Christie M.D., Sun S.S., Ning D.H., Du H., Zhang S.W., Li W.H. (2017). A torsional MRE joint for a C-shaped robotic leg. Smart Mater. Struct..

[36] Ginder J.M., Schlotter W.F., Nichols M.E. Magnetorheological elastomers in tunable vibration absorbers. Proceedings of the SPIE’s 8th Annual International Symposium on Smart Structures and Materials.

[37] Komatsuzaki T., Iwata Y. (2015). Design of a real-time adaptively tuned dynamic vibration absorber with a variable stiffness property using magnetorheological elastomer. Shock Vib..

[38] Komatsuzaki T., Inoue T., Terashima O. (2016). Broadband vibration control of a structure by using a magnetorheological elastomer-based tuned dynamic absorber. Mechatronics.

[39] Park J.E., Lee J., Kim Y.K. (2021). Design of model-free reinforcement learning control for tunable vibration absorber system based on magnetorheological elastomer. Smart Mater. Struct..

[40] Guan X., Zhang J., Li H., Ou J. (2020). Semi-Active Control for Benchmark Building Using Innovative TMD with MRE Isolators. Int. J. Struct. Stab. Dy..

[41] Wang Q., Dong X., Liu L., Yang Q., Ou J. (2020). Wind-induced vibration control of a constructing bridge tower with MRE variable stiffness tuned mass damper. Smart Mater. Struct..

[42] Yang Q., Yang Y., Wang Q., Peng L. (2021). Study on the fluctuating wind responses of constructing bridge towers with magnetorheological elastomer variable stiffness tuned mass damper. J. Intel. Mat. Syst. Str..

[43] Wang B., Tu J., Xu J. (2015). The frequency adjustable tuned mass damper device based on magnetorheological elastomers. Vibroeng. Procedia.

[44] Yang J.N., Lei Y., Pan S., Huang N. (2003). System identification of linear structures based on Hilbert-Huang spectral analysis Part 1: Normal modes. Earthq. Eng. Struct. Dyn..

[45] Yang J.N., Lei Y., Pan S., Huang N. (2003). System identification of linear structures based on Hilbert-Huang spectral analysis Part 2: Complex modes. Earthq. Eng. Struct. Dyn..

[46] Liu T.Y., Chiang W.L., Chen C.W., Hsu W.K., Lin C.W., Chiou D.J., Huang P.C. (2012). Structural system identification for vibration bridges using the Hilbert–Huang transform. J. Vib. Control.

[47] Han J., Zheng P., Wang H. (2014). Structural modal parameter identification and damage diagnosis based on Hilbert-Huang transform. Earthq. Eng. Eng. Vib..

[48] Moncayo H., Marulanda J., Thomson P. (2010). Identification and monitoring of modal parameters in aircraft structures using the Natural Excitation Technique (NExT) combined with the Eigensystem Realization Algorithm (ERA). J. Aerospace Eng..

[49] Seto K. (2006). Vibration Control of Structure.

[50] Li J.F., Gong X.L., Zhang X.Z., Zhang P.Q. (2006). Research on magnetorheological elastomer based on silicone rubber. J. Funct. Mater..

[51] Xu Y., Gong X., Xuan S., Zhang W., Fan Y. (2011). A high-performance magnetorheological material: Preparation, characterization and magnetic-mechanic coupling properties. Soft Matter.

[52] Lynn P.A. (1992). The Discrete and Fast Fourier Transforms.

[53] Liu K., Liang L., Jie L. (2005). Comparison of Two Auto-Tuning Methods for a Variable Stiffness Vibration Absorber. Trans. Can. Soc. Mech. Eng..

[54] James G., Carne T.G., Laufer J. (1993). The Natural Excitation Technique (NExT) for Modal Parameter Extraction from Operating Structures. Int. J. Anal. Exp. Modal Anal..

[55] Huang N.E., Shen S.P. (2005). Hilbert–Huang Transform and Its Applications.

